# Efficacy of Aerobic and Stretching Exercises in Managing Willis-Ekbom Disease (Restless Leg Syndrome) Among Hemodialysis Patients

**DOI:** 10.7759/cureus.71470

**Published:** 2024-10-14

**Authors:** Mohamedalamin Alnoor Altayb Ismail, Islam Daffalla, Taranpreet Singh, Qandeel Rida Siddique, Mohammed Khaleel I. KH. Almadhoun, Rabail Irfan, Moeez Saqib, Muhammad Haris, Zaid Khan, Jaqueline Giselle Farias Fernandes, Asma Iqbal, Syed Faqeer Hussain Bokhari

**Affiliations:** 1 Internal Medicine, Ibrahim Malik Teaching Hospital, Khartoum , SDN; 2 Orthopedics, Cork University Hospital, Cork, IRL; 3 Medicine and Surgery, Mahatma Gandhi Mission (MGM) Medical College, Navi Mumbai, IND; 4 Medicine and Surgery, Amna Inayat Medical College, Lahore, PAK; 5 Medicine and Surgery, Mutah University, Karak, JOR; 6 Pathology, Pak International Medical College, Peshawar, PAK; 7 Internal Medicine, King Edward Medical University, Lahore, PAK; 8 Medicine and Surgery, Lady Reading Hospital, Peshawar, PAK; 9 Medicine, Centro Universitário Cesmac, Maceió, BRA; 10 Medicine and Surgery, King Edward Medical University, Lahore, PAK; 11 Surgery, King Edward Medical University, Lahore, PAK

**Keywords:** aerobic exercise for rls, exercise therapy renal failure, hemodialysis exercise, intradialytic exercise, non-pharmacological rls, non-pharmacological rls treatment, restless legs syndrome (rls), rls management, rls sleep quality improvement, stretching exercises hemodialysis

## Abstract

Restless leg syndrome (RLS) is a neurological disorder characterized by an irresistible urge to move the legs, particularly during rest. It significantly affects the quality of life of hemodialysis patients with high prevalence in this population. While pharmacological treatments, especially dopamine agonists (DAs), are commonly used, they often come with side effects and augmentation phenomena. This systematic review examines the effectiveness of exercise interventions in managing RLS among hemodialysis patients. Eight studies, including randomized controlled trials and quasi-experimental studies, were analyzed. The interventions primarily consisted of aerobic exercises (cycling) and stretching exercises, with durations ranging from eight weeks to six months. The primary outcome measure was RLS severity, assessed using validated scales such as the International Restless Legs Syndrome Study Group (IRLSSG) scale or the Restless Legs Syndrome Questionnaire (RLSQ). Secondary outcomes included sleep quality, depression, and physical performance. The results consistently demonstrated that both aerobic and stretching exercises significantly reduced RLS severity and improved related outcomes. The intradialytic nature of some interventions offered a practical approach to incorporating exercise into patient routines. While exercise showed comparable efficacy to DAs in reducing RLS symptoms and improving depression scores, only DAs significantly enhanced sleep quality in one comparative study. These findings suggest that exercise interventions may be a viable non-pharmacological approach to managing RLS in hemodialysis patients, potentially complementing or reducing reliance on pharmacological treatments.

## Introduction and background

Restless leg syndrome (RLS), also known as Willis-Ekbom disease, is a neurological movement disorder characterized by an overwhelming urge to move the legs, particularly during periods of rest, sitting, or sleep. Patients often experience unpleasant sensations such as tingling, itching, or limb pain, which temporarily improve with movement [1]. RLS often manifests in middle age, though its exact causes remain unclear. A mix of hereditary and environmental factors plays a role, with nearly 40% of cases showing a genetic link [2]. However, RLS is also frequently associated with underlying chronic conditions such as chronic kidney disease, iron deficiency anemia, and Parkinson's disease. Neuropathy, pregnancy, and the use of certain medications like caffeine, calcium channel blockers and lithium worsen symptoms. These diverse factors suggest that RLS can arise from both genetic predisposition and secondary conditions, making it a complex and multifaceted disorder [3].

RLS disrupts sleep and impairs the overall quality of life, especially in populations such as those undergoing hemodialysis for end-stage renal disease (ESRD), where prevalence is notably higher. ESRD patients require replacement therapies to survive, with hemodialysis being one of the most common treatments, affecting over one million people worldwide [4]. However, hemodialysis often comes with various complications, either due to the underlying renal failure or the treatment itself. One prevalent complication is RLS, a sensory-motor disorder marked by an overwhelming urge to move the legs, particularly during rest [5]. The prevalence of RLS in this population is significantly higher than in the general public, estimated between 20% and 80% [4]. This increased prevalence can be attributed to the severe uremia associated with chronic renal failure, which contributes to a complex symptomatology characterized by intermittent and fluctuating symptoms, often leading to delayed diagnosis [5].

Studies suggest that while pharmacological treatments, particularly dopamine agonists (DAs), are the primary therapeutic approach for alleviating RLS symptoms, these medications are not without drawbacks. Side effects and augmentation phenomena are commonly reported, prompting interest in alternative therapies. Exercise has been shown to significantly benefit patients with uremic RLS by improving sleep quality and reducing symptoms such as depression and anxiety. Aerobic exercise, in particular, has been recognized as an efficient and cost-effective strategy to enhance mental well-being and address sleep disturbances in hemodialysis patients [3].

Shahgholian et al. reported that a regimen of stretching exercises over four weeks significantly reduced the severity of RLS symptoms [4]. In contrast, Aliasgharpour et al. found that while initial changes were not statistically meaningful after four weeks, improvements became significant by the eighth week, underscoring the need for longer intervention periods. Furthermore, a six-month intradialytic exercise program was shown to be as effective as low-dosage DA treatment in alleviating RLS symptoms and enhancing depressive states, though only DAs markedly improved sleep quality [6]. Furthermore, reflexology is often used as a complementary therapy to alleviate pain, improve circulation, and enhance relaxation. In patients with chronic conditions like RLS or undergoing treatments such as hemodialysis, reflexology may help reduce discomfort, improve sleep quality, and manage associated symptoms [4].

The variability in the presentation of RLS among renal failure patients complicates management, as symptoms may be intermittent and fluctuating. This highlights the importance of tailored exercise prescriptions to optimize outcomes for this population. Although both DAs and exercise training have demonstrated efficacy in alleviating RLS symptoms, further research is needed to elucidate whether DA treatment can also enhance physical performance and muscle size in patients suffering from uremic RLS. The evolving evidence base underscores the potential of exercise as a complementary intervention in the management of RLS among hemodialysis patients, warranting continued exploration in future studies.

## Review

Materials and methods

This systematic review was conducted to comprehensively assess the effectiveness of exercise interventions in the management of RLS among hemodialysis patients. The review process adhered to the PRISMA (Preferred Reporting Items for Systematic Reviews and Meta-Analyses) 2020 guidelines to ensure a thorough and transparent approach to literature synthesis [7].

Search Strategy

A comprehensive literature search was performed using multiple electronic databases, including MEDLINE (via PubMed), Scopus, Cochrane Central Register of Controlled Trials (CENTRAL), and Web of Science. The search period covered all publications from the inception of each database until July 2024. The following search string was employed: “(exercise OR physical activity OR aerobic OR stretching) AND (Restless Legs Syndrome OR RLS) AND (hemodialysis OR renal failure OR kidney failure OR ESRD OR CKD).” To ensure comprehensive coverage, reference lists from relevant reviews and included studies were manually searched to identify additional studies that may have been overlooked in the initial database search.

Eligibility Criteria

Studies were eligible for inclusion if they met specific criteria related to population, intervention, comparison, outcomes, and study design. The population of interest consisted of adult patients (aged 18 years or older) undergoing hemodialysis with a confirmed diagnosis of RLS, regardless of the duration of RLS symptoms or hemodialysis treatment. The interventions of focus were exercise regimens, including but not limited to aerobic exercises, resistance training, stretching exercises, or intradialytic exercises. The primary outcome of interest was the reduction in RLS severity, as measured by validated scales such as the International Restless Legs Syndrome Study Group (IRLSSG) scale or the Restless Legs Syndrome Questionnaire (RLSQ). Secondary outcomes included sleep quality, quality of life, depressive symptoms, and physical performance. Randomized controlled trials (RCTs) were prioritized for inclusion, though quasi-experimental studies and clinical trials were also considered. Studies published in English were included, while case reports, case series, grey literature, and non-English language articles were excluded from the review.

Study Selection Process

The study selection process was conducted independently by two reviewers. Initially, titles and abstracts of all identified records were screened to exclude studies that did not meet the eligibility criteria. Full-text articles were then obtained and independently reviewed by both reviewers for final inclusion. Any discrepancies between the two reviewers regarding the inclusion of studies were resolved through discussion, with a third reviewer consulted if necessary to reach a consensus.

Data Extraction

Data extraction was carried out using a standardized data collection form. Two reviewers independently extracted data from each included study. The extracted information covered key aspects such as study characteristics, participant demographics, intervention details, comparator details, and outcome measures related to RLS severity, sleep quality, quality of life, depression, and functional capacity.

Data Analysis

Given the narrative nature of this systematic review, a qualitative synthesis of findings was conducted rather than a statistical meta-analysis. The results from individual studies were summarized and integrated to provide a comprehensive overview of the impact of exercise on RLS management in hemodialysis patients. Patterns across studies, consistencies and inconsistencies in findings, and potential factors explaining variations in outcomes were carefully examined. The review also considered the different types of exercise interventions and their respective impact on primary and secondary outcomes.

Results

Study Selection

**Figure 1:**
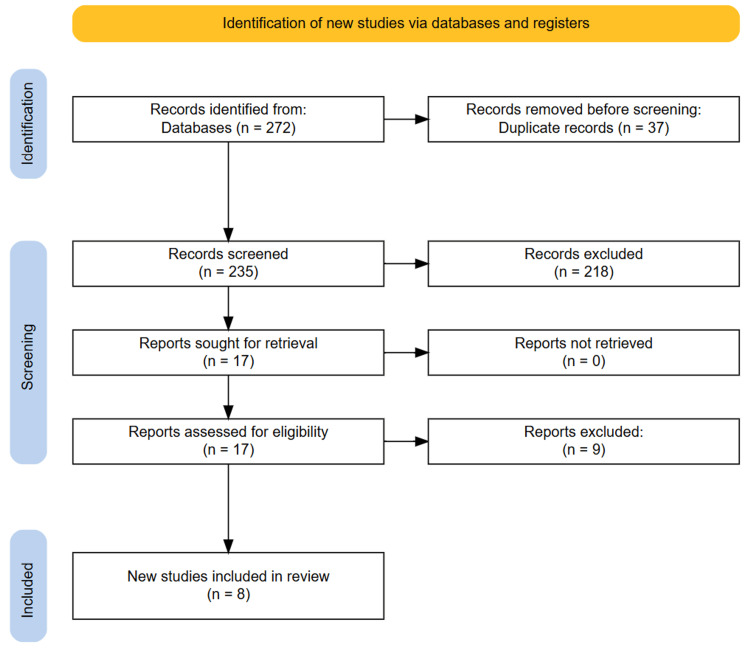
PRISMA diagram illustrating the study selection process. PRISMA: Preferred Reporting Items for Systematic Reviews and Meta-Analyses

The initial database search identified 272 studies. After removing 37 duplicate entries, the titles and abstracts of 235 studies were screened. Of these, 17 studies were deemed potentially relevant based on the inclusion criteria and underwent full-text review. After a detailed evaluation, eight studies were included in the final systematic review. A manual search of the reference lists from the selected studies did not reveal any additional eligible studies. The full study selection process is illustrated in the PRISMA flowchart (Figure 1).

Study Characteristics

This systematic review included eight studies, published between 2013 and 2021, involving a total of 193 hemodialysis patients with RLS. The studies varied in design, comprising RCTs (n = 6) and quasi-experimental studies (n = 2). All studies investigated the efficacy of various exercise interventions in reducing RLS severity and improving other related outcomes such as sleep quality and depressive symptoms in hemodialysis patients. Sample sizes varied across the studies, ranging from 24 to 90 participants. These studies predominantly focused on adults undergoing hemodialysis for chronic renal failure. The inclusion of patients with confirmed RLS diagnoses ensured that the outcomes were directly related to the management of RLS symptoms in this specific population. All the participants across the eight studies were adults aged 18 years or older. The studies included both male and female participants, with no specific emphasis on gender differences. Four studies (RCTs) studied the effect of aerobic exercises while four studies (two RCTs and two quasi-experimental) studied the effect of stretching exercises. The length of the interventions varied across the studies, ranging from eight weeks to six months.

Aerobic exercises involved bicycle pedaling. Giannaki et al. conducted a study where participants cycled on a recumbent ergometer at 60-65% of their maximal capacity during hemodialysis sessions [8,9]. This exercise was performed three times per week for six months, with intensity adjusted monthly based on individual performance. Mortazavi et al. similarly used a cycling intervention, but for shorter sessions (20 minutes of cycling per session) over 16 weeks [3]. The stretching interventions targeting the lower limbs, including hamstring, quadriceps, and gluteal stretches, among others, typically lasted 15 to 40 minutes per session, with two to three sessions per week over a span of 8 to 20 weeks. Giannaki et al. also incorporated resistance training into their aerobic exercise regimen, progressively increasing resistance during cycling [8]. This combined approach aimed to improve not only RLS symptoms but also muscle strength and physical performance, which are often compromised in hemodialysis patients due to muscle wasting.

Each study included a control or comparator group, which either received no exercise intervention or underwent standard care. In some studies, control groups engaged in warm-up exercises that were significantly less intense than the intervention, serving as a low-intensity comparison [3,10]. Giannaki et al. compared aerobic exercise to both a placebo and DA, a pharmacological treatment for RLS [9]. This allowed for a more nuanced comparison between exercise and conventional pharmacotherapy. The primary outcome of interest in all studies was the severity of RLS symptoms, typically measured using the IRLSSG scale or the RLSQ. These validated scales assess the frequency and intensity of RLS symptoms, providing a standardized way to compare outcomes across different interventions. Secondary outcomes included sleep quality, depression, and physical performance. 

The results across all eight studies were generally positive, indicating that exercise interventions significantly reduced RLS severity and improved related outcomes in hemodialysis patients. For instance, Giannaki et al. reported a 58% reduction in RLS severity in the aerobic exercise group, along with improvements in sleep quality, depression scores, and physical performance [8]. Similarly, Aliasgharpour et al. found that stretching exercises significantly reduced RLS severity compared to the control group, with effects becoming more pronounced after eight weeks of intervention [6]. Fauzi and Triaswati observed significant improvements in both RLS severity and sleep quality in the intervention group after eight weeks of stretching exercises [10]. In contrast, the control group, which performed warm-up exercises, showed no significant changes in either outcome. The results are summarized in Table 1.

**Table 1:** Summary of the results of included studies. RLS: Restless Leg Syndrome; HD: Hemodialysis; IRLSSG: International Restless Legs Syndrome Study Group; PSQI: Pittsburgh Sleep Quality Index; RLSQ: Restless Legs Syndrome Questionnaire; SF-63: Short Form-63 (Quality of Life Questionnaire)

Author	Year	Study Design	Sample Size	Population Characteristics	Intervention	Type of Exercise	Comparator	RLS Severity Scale	Outcome Measures	Duration of Intervention	Main Results
Fauzi and Triaswati [10]	2021	Quasi-experimental study	38 (19 intervention, 12 control)	Adult patients undergoing chronic hemodialysis for at least three months	Stretching exercises	Ankle plantar flexion, gastrocnemius stretch, soleus stretch, hamstrings stretch, and quadriceps stretch	Warm-up exercises (control group)	IRLSSG Scale	Severity of RLS symptoms, Sleep quality (PSQI)	Eight weeks (two times/week, 15 minutes/session)	Significant reduction in RLS severity in the intervention group (p < .001)
Shahgholian et al. [4]	2016	Randomized clinical trial	90	Hemodialysis patients with RLS	Stretching exercises	Not mentioned	Reflexology and control group	RLSQ	Severity of RLS symptoms	Four weeks (three sessions/week, 30-40 min each)	Both reflexology and stretching exercises significantly reduced the severity of restless leg syndrome compared to the control group (p < 0.001). There was no significant difference between reflexology and stretching exercises groups.
Algendy and Bahgat [11]	2019	Quasi-experimental	40 (20 intervention, 20 control)	Hemodialysis patients with RLS	Stretching exercises	Toe touch, standing foot grab, kneeling adductor stretch, wall hamstring stretch, bench hip flexor stretch, it band stretch, lateral lunge and half-kneeling calf stretch	Standard care	IRLSSG Scale	Severity of RLS symptoms	20 weeks (two times/day, 30 minutes/session)	Significant improvement in RLS severity in the intervention group compared to the control group post-intervention.
Aliasgharpour et al. [6]	2016	Randomized controlled trial	33 (17 intervention, 16 control)	Hemodialysis patients with RLS	Stretching exercises	Hip rotation; quadriceps, knee-to-chest, hamstring and gluteal stretches, straight leg raises, and side-lying leg lifts, with each muscle stretched for three sets of 10 repetitions, holding for 5 seconds per stretch.	Standard care	IRLSSG Scale	Severity of RLS symptoms	Eight weeks (three times/week, 30 minutes/session)	Stretching exercises significantly reduced RLS severity scores in the intervention group compared to the control group (p < 0.001).
Giannaki et al. [8]	2013	Single-blind randomized controlled trial	24 (12 intervention, 12 control)	Hemodialysis patients with RLS	Aerobic exercises with progressive resistance	Cycling on a recumbent ergometer at 60-65% of maximal capacity during HD sessions with intensity readjusted monthly. The same exercise in the control group but without resistance	Exercise with no resistance	IRLSSG Scale	RLS severity, sleep quality, depression, functional capacity	Six months (three times/week, 45 minutes/session)	RLS severity declined by 58% in the exercise group (p=0.003), no significant change in the control group. Sleep quality improved in the exercise group (p=0.038). Depression score improved in the exercise group (p=0.000). Functional capacity improved in the exercise group No adverse effects or augmentation phenomena reported
Giannaki et al. [9]	2013	Randomized, partially double-blind, placebo-controlled trial	32 (16 exercise group, 8 dopamine agonist group, 8 placebo)	Hemodialysis patients with RLS	Aerobic exercises with progressive resistance	Cycling on a recumbent ergometer at 60-65% of maximal capacity during HD sessions with intensity readjusted monthly	Dopamine agonist and Placebo	IRLSSG Scale	RLS severity, sleep quality, depression, functional capacity	Six months (three times/week)	Both exercise training and dopamine agonists significantly reduced RLS symptoms (46% and 54% respectively) and improved depression scores. ​ Exercise training improved lean body mass, reduced fat infiltration in muscles, and enhanced physical performance. Only the dopamine agonists significantly improved sleep quality (P = 0.009). No significant side effects were reported in either treatment group.
Mortazavi et al. [3]	2013	Randomized clinical trial	26 (13 intervention, 13 control)	Hemodialysis patients with RLS	Aerobic exercise	First five minutes for warm-up followed by bicycle pedaling for 20 minutes, and finally five minutes of cool-down	Standard care	RLSQ	RLS severity, Quality of life (SF-63)	16 weeks (three times/week, 30 minutes/session)	Significant improvement in RLS severity in the exercise group compared to the control group (mean difference: -5.5 ± 4.96 vs -0.53 ± 2.3, p=0.003). No significant difference in quality of life between groups

Discussion

This systematic review of eight studies investigating the effectiveness of exercise interventions in managing RLS among hemodialysis patients yields promising results. The findings consistently demonstrate that both aerobic and stretching exercises can significantly reduce RLS severity and improve related outcomes such as sleep quality, depression, and physical performance. The positive outcomes observed across the included studies suggest that exercise interventions may be a viable non-pharmacological approach to managing RLS in hemodialysis patients. The reduction in RLS severity reported by Giannaki et al. (58% decrease) and the significant improvements noted in other studies underscore the potential of exercise as a therapeutic tool [8-12]. These findings are particularly noteworthy given the high prevalence of RLS in hemodialysis patients and the limitations of pharmacological treatments.

The effectiveness of both aerobic and stretching exercises indicates that different types of physical activity can be beneficial. This flexibility in the intervention type is advantageous, as it allows for tailoring exercise programs to individual patient preferences and capabilities. The intradialytic nature of some interventions, such as cycling during hemodialysis sessions, offers a practical approach to incorporating exercise into patients' routines without additional time commitments [8,9]. The results align with previous research on exercise interventions for RLS in the general population. A meta-analysis by Song et al. found that exercise training significantly improved RLS severity, depression, and sleep quality in hemodialysis patients [5]. Our review extends these findings by including more recent studies and providing a detailed analysis of different exercise modalities. The comparison of exercise with DAs in one study is particularly intriguing [9]. While both interventions reduced RLS symptoms and improved depression scores, only DAs significantly enhanced sleep quality. This suggests that while exercise may be an effective alternative to pharmacological treatment for some aspects of RLS management, a combination of approaches might be optimal for addressing all symptoms [13].

The underlying mechanisms by which exercise alleviates RLS symptoms in hemodialysis patients warrant further exploration. Several hypotheses can be proposed to explain this effect. First, exercise is known to improve circulation, potentially addressing peripheral hypoxia, which is believed to contribute to RLS symptoms by enhancing blood flow [14]. Second, physical activity is thought to modulate neurotransmitter systems, particularly dopamine and opioid pathways, both of which are implicated in the pathophysiology of RLS [15,16]. Additionally, exercise may aid in the reduction of uremic toxins, which are common in hemodialysis patients and may play a role in the development of RLS. Another possible mechanism is the psychological benefit of exercise, as improvements in depression scores suggest that the mental health effects of physical activity may also contribute to symptom relief [17]. Finally, muscle conditioning through strengthening and stretching exercises could reduce muscle tension and discomfort, helping to alleviate some of the physical symptoms associated with RLS. Together, these mechanisms highlight the multifaceted role of exercise in managing RLS in this population [18].

The findings of this review carry several important implications for the management of RLS in hemodialysis patients. First, there is a compelling case for the integration of structured exercise programs within hemodialysis units, potentially during dialysis sessions. This approach could help ensure that patients receive regular physical activity, which has been shown to alleviate RLS symptoms. Second, a personalized approach to exercise interventions is warranted; given the demonstrated efficacy of both aerobic and stretching exercises, clinicians should tailor these programs to align with individual patient preferences and physical capabilities, enhancing adherence and effectiveness [19]. Additionally, exercise should be viewed as a complementary therapy alongside pharmacological treatments. This integration could potentially allow for reduced dosages of medications, minimizing the risk of side effects associated with pharmacological interventions. The sustained benefits observed in longer exercise interventions underscore the importance of ongoing programs for the long-term management of RLS, suggesting that consistent physical activity should be a fundamental component of care for these patients [8,9,20]. Finally, the improvements noted in secondary outcomes, such as sleep quality and depressive symptoms, highlight the potential of exercise to address multiple dimensions of patient well-being [8-10]. This holistic approach to care not only aims to alleviate RLS symptoms but also fosters overall health and quality of life, underscoring the need for healthcare providers to prioritize physical activity in treatment plans for hemodialysis patients suffering from RLS.

Despite the promising results, several limitations in the existing research need to be addressed. First, the small sample sizes in most studies limit the generalizability of the findings. Larger, multi-center trials are necessary to confirm the observed benefits of exercise in managing RLS in hemodialysis patients. Second, although some studies extended up to six months, longer-term investigations are essential to determine the sustainability of the benefits and the potential long-term effects of exercise on RLS progression. A third limitation is the variability in exercise protocols across the studies, which makes it difficult to draw consistent conclusions about the most effective type or intensity of exercise. Future research should focus on establishing standardized, evidence-based exercise regimens for RLS management in this patient population. Additionally, more studies are needed to explore the physiological mechanisms underlying the benefits of exercise in RLS, which could inform more targeted and optimized interventions. Another area requiring attention is the comparative effectiveness of exercise versus pharmacological treatments. Only one study in this review directly compared exercise with medication, limiting our ability to assess the relative benefits of these two approaches [9]. Future research should include studies that compare exercise with standard pharmacological therapies, as well as investigate the effects of combining exercise and medication. Adherence to exercise programs is another critical factor that needs further exploration. Understanding the barriers and facilitators to long-term engagement with exercise will be vital for maximizing the clinical benefits of this intervention. Lastly, while the included studies showed improvements in RLS symptoms and secondary outcomes such as sleep quality and depression, more comprehensive evaluations of how these improvements translate into overall quality of life are necessary.

## Conclusions

This systematic review provides compelling evidence for the effectiveness of exercise interventions in managing RLS among hemodialysis patients. Both aerobic and stretching exercises demonstrated significant benefits in reducing RLS severity and improving related outcomes such as depression and physical performance. The flexibility in intervention types allows for personalized exercise programs tailored to individual patient needs and capabilities. The integration of structured exercise programs within hemodialysis units, potentially during dialysis sessions, could be a practical approach to ensure regular physical activity for these patients. While exercise shows promise as a non-pharmacological treatment option, a combination of exercise and pharmacological approaches might be optimal for comprehensive symptom management. Future research should focus on larger, long-term studies to confirm these findings, standardize exercise protocols, and explore the physiological mechanisms underlying the benefits of exercise in RLS. Additionally, comparative studies between exercise and pharmacological treatments, as well as investigations into adherence factors, are needed to optimize the clinical application of exercise interventions for RLS in hemodialysis patients.
